# Evaluating the Implementation of Home-Based Sexual Health Care Among Men Who Have Sex with Men: Limburg4zero

**DOI:** 10.1007/s10461-024-04579-6

**Published:** 2025-01-08

**Authors:** Cornelia Johanna Dorothy Goense, Ymke J. Evers, Jonas Manait, Christian J. P. A. Hoebe, Inge H. M. van Loo, Dirk Posthouwer, Robin Ackens, Roland van Hooren, Rocxanne Theuerzeit, Rik Crutzen, Sarah E. Stutterheim, Nicole H. T. M. Dukers-Muijrers

**Affiliations:** 1 Department of Social Medicine, Care and Public Health Research Institute (CAPHRI), Maastricht University, P.O. Box 616, 6200 MD Maastricht, The Netherlands; 2 Department of Sexual Health, Infectious Diseases and Environmental Health, Living Lab Public Health Mosa, Public Health Service South Limburg, Heerlen, The Netherlands; 3 Department of Health Promotion, Care and Public Health Research Institute (CAPHRI), Maastricht University, Maastricht, The Netherlands; 4 Department of Medical Microbiology, Infectious Diseases and Infection Prevention, Care and Public Health Research Institute (CAPHRI), Maastricht University Medical Center+ (MUMC+), Maastricht, The Netherlands; 5 Division of Infectious Diseases, Department of Internal Medicine, Maastricht University Medical Center (MUMC+), Maastricht, The Netherlands; 6 Department of Integrated Care, Maastricht University Medical Center+ (MUMC+), Maastricht, The Netherlands

**Keywords:** Home care services, STI testing, HIV testing, Home-based sexual health care, Self-sampling, MSM, Implementation science

## Abstract

Home-based sexual health care (including self-sampling testing) could reduce barriers to clinic-based testing. This study systematically evaluated the implementation of home-based sexual health care (‘Limburg4Zero’) among men who have sex with men (MSM) in a mixed urban–rural region of the Netherlands. We systematically assessed implementation outcomes (contextual domains, population reached, effectiveness, adoption by health care providers (HCP), implementation fidelity, and maintenance) using the practical, robust implementation and sustainability model. We used a mixed-methods research design that combined survey and in-depth interview data from our priority population (MSM) and implementers with insights from our community advisory board and field notes from monthly meetings with the implementers. Of 1076 requested tests, 906 MSM received a (self-sampling STI/HIV) test kit and sexual health information. Most participants (median age 31) had a university or college degree and had a Western ethnicity. The return rate for laboratory testing was 67%. Home-based care participants more often never tested HIV before (39%) compared to STI clinic attendees (28%). Adoption by HCP was highly acceptable, although perceived pragmatic and technical challenges were experienced. Key elements of the home-based care service were implemented as designed, with co-created adjustments for optimization during the implementation. Maintaining the innovation in the long term was desirable, although future recommendations for efficient continuation should be considered. Implementation of home-based sexual health care has demonstrably increased care accessibility for MSM who had never previously been tested. Therefore, a systematic approach is crucial to foster the impact of home-based sexual health care.

**Supplementary Information:**

The online version contains supplementary material available at 10.1007/s10461-024-04579-6.

## Introduction

Sexually transmitted infections (STI) and human immunodeficiency virus (HIV) continue to impact global public health. Worldwide, more than one million new HIV are diagnosed each year, especially among key populations such as men who have sex with men (MSM). The World Health Organization (WHO) has therefore formulated strategic goals designed to end the HIV epidemic by 2030 [1]. These UNAIDS goals are set to the ‘95-95-95’ target, which signifies that 95% of all people with HIV are diagnosed, 95% receive antiretroviral therapy (ART), and 95% achieve viral suppression [2]. In addition, the European Centre for Disease Prevention and Control (ECDC) reports a rise in STI cases, including syphilis, *Neisseria gonorrhoeae* (NG), and *Chlamydia trachomatis* (CT), across Europe [3].

Effective strategies for reducing transmission are regular STI and HIV testing, timely treatment, and use of pre-exposure prophylaxis (PrEP). In the Netherlands, the Amsterdam area was approaching zero new HIV diagnoses in 2023 [4]. Public health and clinical practice guidelines in the Netherlands recommend that key populations (e.g., MSM) test for HIV and other STI at least every six months [5]. However, in the Netherlands, Stichting HIV monitoring (SHM) estimated that approximately 1,390 individuals are not yet aware of their HIV status. Currently, most new HIV are diagnosed among MSM [6]. Demonstrated barriers to clinic-based testing include anticipated stigma, waiting times, and transportation constraints—which are more prevalent for people living in less urban areas [7, 8].

Testing or sampling outside of a clinic can contribute to a greater accessibility for an extended reach to, at-risk and untested key populations [9, 10]. WHO recommended the implementation of self-care interventions for STI and HIV testing to promote self-control and autonomy in sexual health [11]. Such home-based sexual health care ideally is integrated with sexual health care consisting of STI and HIV self-sampling testing (i.e., self-sampled and sent to the laboratory for testing) and additional sexual health information, counseling, and appropriate treatment [12, 13]. Successful implementation may be characterized by increased uptake of STI and HIV testing by the priority population (here: previously untested MSM).

Several factors might affect the implementation process, such as individual beliefs and contextual factors. Health care providers (HCP) serve a key role as implementers in the success of a home-based sexual health care service because its implementation success anticipates the ability, capability, and willingness of implementers to engage in the intervention [14]. Home-based sexual health care is a complex intervention because it requires the collaboration of different parties to successfully implement the innovation in practice. Therefore, a systematic framework may be useful for the guidance of an implementation process from development to evaluation [15].

### Objective

Recently, the Center for Sexual Health of the Public Health Services in Limburg (the Netherlands) developed ‘Limburg4zero’; an integrated home-based sexual health care program including self-sampling STI and HIV testing and online sexual health information and counseling by the Center for Sexual Health [16]. The intervention was developed by using the Intervention Mapping (IM) approach for systematic intervention design, evidence-based, regionally tailored, systematically designed, and developed in co-creation with users and implementers [17]. This study aimed to systematically evaluate the implementation of Limburg4zero using the practical, robust implementation and sustainability model (PRISM) [18].

## Methods

### Intervention Description

The Center for Sexual Health, previously known as the STI clinic, delivers free-of-charge sexual health care to key populations in the Netherlands. As of April 2021, STI clinics in Limburg (southeast of the Netherlands) initiated the implementation of free-of-charge, home-based sexual health care for MSM, including STI/HIV testing via self-sampling and tailored sexual health information (Limburg4zero) [16]. A planning group was responsible for the implementation of this home-based care innovation, which consisted of STI clinic HCPs (nurses, doctors), laboratory professionals, researchers, policymakers, and other regional stakeholders. The implementation script that was developed by this planning group was used to establish a complete picture of the designed processes. This script contains several elements such as planning group construction, a step-by-step plan on how to use the intervention in practice, responsibilities, and weekly planning, monitoring, and evaluation approach. Additionally, a community advisory board (CAB) was recruited that suggested strategies for optimization of home-based sexual health care among other topics. Members were MSM residing in Limburg, and were recruited from STI clinic attends, participating in previous research, or participating in home-based sexual health care.

#### Phased Implementation and Communication

**Fig. 1:**
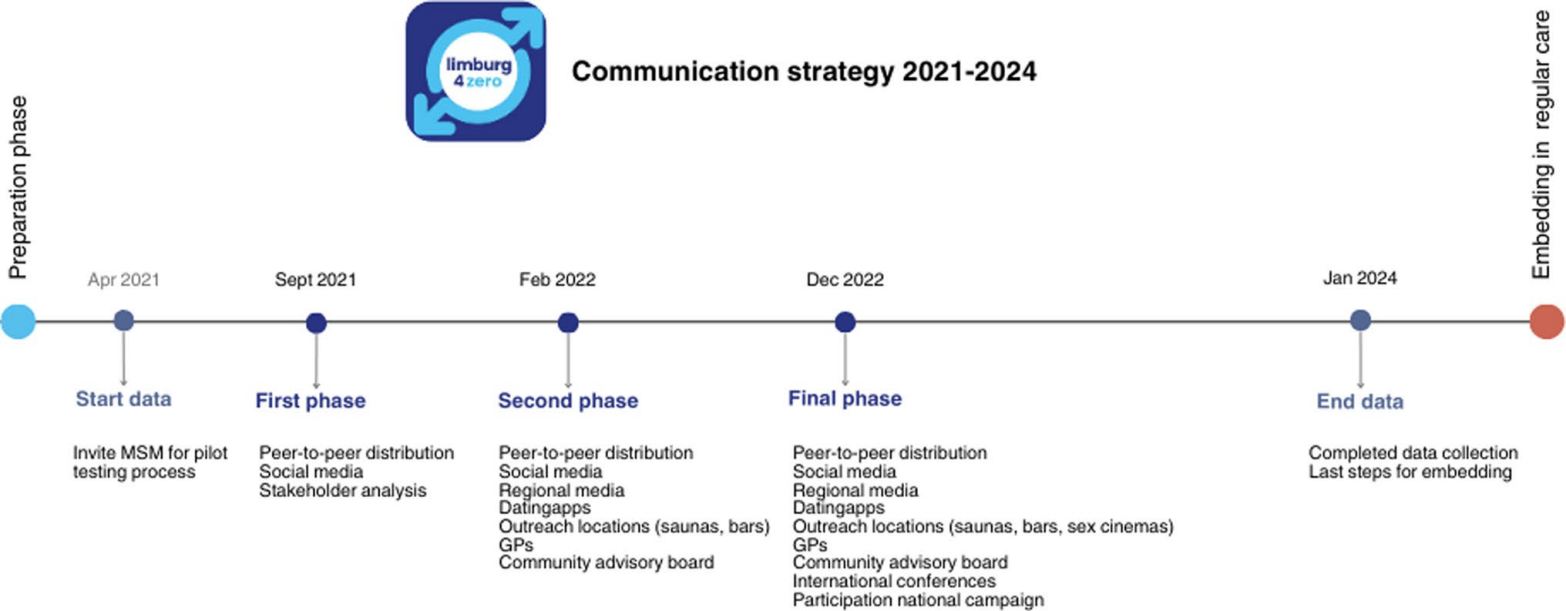
Communication strategy considering communicational methods of Limburg4zero from April 2021 to January 2024

Within the planning group, we agreed upon phased communication, starting with a pilot (n = 20) in which invited MSM tested the implementation process. In the following phases, we gradually added more methods of communication. Via interviews with HCP and CAB meetings, we identified important meeting places and online channels to reach previously untested MSM in our region. Figure 1 shows the stepped approach to communication. Tailored communication messages were co-created with the CAB to target the identified behavioral determinants. For example, attitude and self-efficacy were targeted with the message: *“Safe and confidential testing for HIV and STI, where and when it suits you?”* and subjective norm with *“Check yourself for STI and HIV. Protect others.”* (see Supplementary material S4 which displays an example of communication material). Given that we equipped all communication materials and channels in the last implementation phase, our evaluation focused solely on the communication from that phase (January 1 to December 31, 2023).

#### Application Home-Based Sexual Health Care Service

Potential participants could apply for the self-sampling STI and HIV test kit on the program’s website. Participants were eligible to apply for a test if they were aged over 16 years, lived in the province of Limburg (based on postal code), identified as male, and previously had sex with men. The test kit was available to MSM, who had reportedly not previously tested HIV positive, and not used PrEP. In the process of applying, potential participants were asked to respond to several questions that queried social demographic variables, and sexual history. Based on their responses, participants received tailored sexual health information (e.g., when they were not vaccinated for Hepatitis B, they received information on how to vaccinate for Hepatitis B) [16]. At home self-collected urine, anorectal and oropharyngeal swabs were taken for CT and NG testing. Capillary blood was self-collected in a tube via finger-prick for syphilis and HIV testing. Participants sent collected samples to the medical microbiology laboratory. Testing materials were labeled with a code, accessible only to designated STI clinic staff for linking to patient information. When a positive result was obtained, HCP invited the participant to the clinic for counseling and treatment, specifically for CT and NG. If the test indicated reactive syphilis or HIV, confirmatory testing was conducted. In the event of a confirmed positive HIV result, participants were invited for counseling and referred to an HIV treatment center (see Supplementary material S1 where the complete client pathway is shown).

#### Laboratory Procedures

For CT and NG testing, standard-of-care polymerase chain reaction (PCR) tests were applied (Roche Cobas 4800, Roche Diagnostics, Switzerland). A laboratory assistant manually divided the blood sample in preparation for to test. A laboratory algorithm was applied, depending on the amount of blood collected. When serum volume was over 200 µl standard-of-care, HIV and syphilis tests were performed (Elecsys HIV Duo and Elecsys Syphilis, Roche Diagnostics, Germany). When less blood was collected, rapid HIV and syphilis tests were used (Alere™ SD Bioline HIV/Syphilis Duo, Abbott, USA), preferably with additional quantitative *Treponema pallidum* particle agglutination assay (TPPA, Fujirebio, Japan). For confirmatory (clinic-based) HIV testing an immunoblot was used (Geenius HIV-1/2 confirmatory assay, Bio-Rad, USA), and a quantitative Rapid Plasma Reagin (Carbogen® RPR, Switzerland) for confirmatory syphilis testing. Laboratory results were returned to the STI clinic by referencing the code labeled on the testing materials.

### Practical, Robust, Implementation and Sustainability Model (PRISM)

We systematically evaluated the implementation of home-based sexual health care by following PRISM. This framework consists of six elements, that is contextual domains, reach, effectiveness, adoption, implementation, and maintenance [18]. Table 1 shows the operationalization of the different PRISM constructs, indicators, and measures. In the contextual domains, external elements such as policy, epidemiology, implementation, and sustainability infrastructure were described. Consequently, the proportion of the key population reached with home-based sexual health care compared to clinic attendees, and the effectiveness in reaching untested individuals were discussed. Within the adoption element, the barriers and facilitators for HCP to adopt the innovation were explored. This was followed by an examination of the implementation fidelity, assessing to what extent the key elements were implemented as designed. Finally, recommendations were made for the maintenance of home-based sexual health care in addition to regular sexual health care.

**Table 1:** Operationalization of contextual domains, reach, effectiveness, adoption, implementation, and maintenance outcomes following PRISM

Dimension	Constructs	Indicators	Data source
Contextual domains	Intervention description	Description of intervention content	Implementation script Field notes of monthly meetings
	Implementation and sustainability infrastructure	Planning group characteristics (profession, role in implementation), communication, training, and support implementers, plan for sustainability	Implementation script Field notes of monthly meetings
	External environment	Epidemiology of HIV in Limburg	Data of people with HIV in Limburg provided by Stichting HIV Monitoring (SHM)
Outcomes			
Reach (R)	Participation criteria	Triage questions (MSM without HIV, no PrEP use, living in the postal area, no reason for clinic invite)	Test kit orders questionnaire
	Percent individuals who participate	% Sent test kits	Test kit orders questionnaire
	Characteristics of participants compared to non-participants or to target population	Characteristics (age, education, migrant background, urbanization, sexual behavior, HIV test behavior) of people who ordered but did not return the kit vs. those who did	Test kit orders questionnaire
	Use of qualitative methods to understand reach and recruitment	Which communication channels used Feedback on the availability of the test kit	Test kit orders questionnaire CAB meetings MSM interviews
Effectiveness (E)	Measure of primary outcome	% previously untested or irregularly tested subgroups	Test kit orders questionnaire
	Measure of broader outcomes	% education, % age groups, % urban/non- urban, % sexual risk behavior, % sexual orientation, % migrant background; % STI/HIV detection	Test kit orders questionnaire
	Measure of robustness across subgroups	Characteristics of test kit users vs. clinic attendees	Test kit orders questionnaire Electronic patient registry of the STI clinic
	Measure of short-term attrition (%) and differential rates by patient characteristics or treatment condition	Cross table previously untested vs. previously tested by characteristics (age, education, level of urbanization, sexual risk behavior)	Test kit orders questionnaire
	Measure of user acceptability	Difficulty in performing the tests, willingness to use the innovation in the future, recommendation of the innovation to others	Evaluation questionnaire
Adoption (A)	Percent of staff invited that participate	Planning group characteristics (profession, role in implementation)	Field notes of monthly meetings
	Use of qualitative methods to understand staff participation	Acceptability of implementers to adopt the care innovation (attitude, self-efficacy, intention (TPB), barriers, facilitators (HB))	Semi-structured in-depth interviews with HCP
Implementation (I)	Percent of “perfect” intervention delivery completed	List of key elements of the intervention and check how many were implemented as scripted	Implementation script
	Adaptations made to intervention during the study	List of key elements that were adjusted or added to the process	Field notes of monthly meetings
	Cost of intervention	Expected effort and time from HCP to implement this care innovation	Semi-structured in-depth interviews with HCP
	Consistency of implementation across staff/time/settings/subgroups (not about differential outcomes, but a process)	List of key elements with their implementation status (implemented as scripted, implemented but adjusted, not yet)	Implementation script Field notes of monthly meetings
	Use of qualitative methods to understand implementation	Acceptability of implementers to adopt the care innovation (attitude, self-efficacy, intention, barriers, and facilitators)	Monthly meetings Semi-structured in-depth interviews with HCP CAB meetings MSM interviews
Maintenance (M)	*Individual level* Recommendation for maintaining primary and broader outcomes at ≥ 6mo follow-up after final intervention contact	Willingness to use the test kit in the future, in-depth information, and suggestions on maintenance of home-based sexual health care in the future	Evaluation questionnaire CAB meetings MSM interviews
	*Organizational level* Recommendation for maintaining program at ≥ 6 month post-study funding and long- term adaptation	Willingness to adopt the innovation in the future, in-depth information, and suggestions on maintenance of home- based sexual health care in the future	Monthly meetings Semi-structured in-depth interviews with HCP Evaluation meeting

### Study Design

We used a mixed-method study design to evaluate the implementation success of home-based sexual health care in Limburg4zero. Successes were characterized by reaching vulnerable MSM who have not previously accessed sexual health care for testing HIV.

#### Quantitative Methods

As shown in Table 1, several quantitative data sources were used, including regional epidemiological data, the online test kit orders questionnaire, STI questionnaire data of clinic attendees, and the evaluation questionnaire among test kit users. Regional epidemiological data included data on people with HIV in the Limburg region (externally provided data). The online test kit orders questionnaire examined participants’ sexual (risk) behavior, HIV test behavior, PrEP use, chemsex, and sociodemographic characteristics (see Supplementary material S2, the online test kit orders questionnaire). In comparison, we analyzed the same data of all MSM without HIV who did not use PrEP and who attended the clinic in the same period from the electronic patient registry of the STI clinic (n = 4356). The evaluation questionnaire was distributed among those who received results of the self-sampling STI/HIV tests, considering their experience with the process of home-based sexual health care (see Supplementary material S3 for the evaluation questionnaire). Participants signed an informed consent before participating in the evaluation questionnaire.

#### Measures

HIV test behavior was measured by the frequency and location of last HIV test. New diagnoses of STI and HIV were assessed in percentages confirmed CT, NG, syphilis, and HIV diagnoses. Acceptability of the test kit was measured in terms of difficulty performing the tests, willingness to use the test kit in the future, whether people would recommend the test kit to others, and how they would appraise the test kit. The difficulty of performing different tests (blood, urine, swabs) was examined on a scale from 0 (easy) to 100 (difficult), and willingness was measured with a 5-point Likert scale (completely disagree–completely agree) by asking ‘Would you use the test kit again the future?’. Recommendation to others was based on the Net Promotor Score (NPS), where scores 9 and 10 (scale 1–10) reflected the ‘promotors’ of the test kit [19]. Lastly, we asked the participants to grade the test kit on a scale from 1 (very poor) to 10 (excellent). There was room for additional open-ended remarks. Sociodemographic characteristics such as level of urbanization and ethnicity were based on definitions of the Central Bureau of Statistics in the Netherlands (https://www.cbs.nl/). Urbanized is defined as more than 1500 addresses per km^2^, and non-urban is defined as less than 1500 addresses per km^2^. Western ethnicity indicated being born in Europe (except Turkey), North America, Oceania, Indonesia or Japan. Non-western ethnicity indicates the participant or at least one parent being born in Africa, Latin America Asia (except Indonesia and Japan), or Turkey. Education was categorized as college or university, vocational training, and less than high school. Sexual (risk) behavior was defined by gender of sex partners, condomless anal intercourse (CAI) with any partner, and chemsex. Chemsex was defined as having used one of the following drugs before or during sex in the past six months: *crystal meth, cocaine, 2-CB, 3MMC, 4-FA, 4-MEC, GHB, GBL, ketamine, MDMA, mephedrone, speed and XTC* [20].

#### Statistical Analyses

Descriptive statistics provided insights into sexual (risk) behavior, testing behavior, the evaluation of home-based sexual health care, and sociodemographic characteristics. Sexual behavior, testing behavior, and sociodemographic characteristics were compared between STI clinic attendees and home-based care participants. Comparisons were also made between home-based care participants who returned the test kit and those who did not, as well as between previously tested and untested subgroups. Chi-square tests were used for these comparisons. All quantitative data were descriptively analyzed with IBM SPSS statistics V26 (IBM Corp., Armonk, N.Y., USA).

#### Qualitative Methods

From May 2021 to September 2021, we conducted semi-structured in-depth interviews among STI clinic HCPs (n = 7) and general practitioners (GPs) (n = 7). STI clinic HCPs were recruited through e-mail invitation, and GPs were recruited by e-mail, phone call, and snowball sampling (see Supplementary material S5, which shows the characteristics of participants). Participants signed an informed consent regarding the use and analysis of interview data. We explored facilitators and barriers regarding adoption and implementation of home-based sexual health care. In addition, field notes from monthly planning group meetings with the planning group during the adoption and implementation process were evaluated. We also evaluated notes from two CAB meetings and six one-on-one interviews with MSM. Here, an informed consent was signed by MSM before participating in the CAB meeting and interviews.

#### Topics

The interview guide was developed based on the diffusion of innovation theory (DOI) and the measurement instrument for determinants of innovations (MIDI) [21, 22]. We explored facilitators and barriers associated with the adoption of home-based sexual health care by HCPs. Additionally, we analyzed determinants of innovation and organizational factors concerning the implementation process.

#### Analyses

In-depth semi-structured interviews with HCP were transcribed verbatim by a Dutch organization specialized in transcriptions. The Rapid Assessment Procedure guided the coding and analysis process, resulting in the optimization of the implementation process. This method is used for gathering and analyzing qualitative data to inform decision-making, often used in health and development fields to quickly understand community needs and contexts. Thematic analysis was used with a combination of inductive and deductive coding. We included interviews, focus groups, and other observations (i.e., field notes) [23]. Data coding was performed and checked by the first three authors. Disagreements were resolved through mutual agreement between the first three, and the last author. For coding, we used qualitative research software ATLAS.ti 22.1.5.0.

## Results

### Contextual Domains

#### External Environment

In 2020 the Netherlands introduced a national program ‘NL to 0’, to reduce new HIV diagnoses to zero. [24]. A regional approach was encouraged, and funding was available to support regions towards zero new HIV. Therefore, in the Limburg region, we aimed to increase the accessibility of sexual health care and implement regional home-based sexual health care for MSM (Limburg4zero).

Currently, in the Limburg region, of the 93% of HIV-diagnosed people, 87% were on antiretroviral therapy, and 83% had a suppressed viral load. An estimated 70 (range 50–120) remained undiagnosed (7%). In the past 10 years, there were 378 new HIV diagnoses, most of them among MSM (69.8%; 264/378).

#### Implementation and Sustainability Infrastructure

The planning group evaluated the progress of implementation in monthly meetings on three domains (1) intervention and logistic implementation, (2) research, and (3) communication. Whereas policy offers, managers, and researchers were mostly responsible for monitoring and developing the implementation as designed, nurses and doctors were responsible for the execution of the project and client handling. All group members were responsible for identifying areas for optimization and setting clear roles and tasks to carry out these optimization steps (cyclic optimization). The implementation script was updated continuously throughout the implementation process by adjustments that emerged from the monthly meetings. Examples would be optimizing laboratory algorithms, simplifying the registration process, or expanding communicational efforts. Collaboration with external stakeholders (i.e., community-based organizations or outreach locations) was most important to maintain the best fit and accessibility to the priority population (reach).

### Reach

#### Communication Strategy

Figure 1 shows the communication strategy from 2021 to the start of 2024, including the use of different methods and channels over time, adding channels progressively. In 2023, participants reported having accessed the test application website via the general STI clinic website, (22.5%, 119/529), STI clinic location (18.3%, 97/529) via peers (13.0%, 69/529), and social media (such as an informative website for MSM and dating sites or apps) (11.4%, 47/529). Open-ended responses indicated additional channels such as online through search engines or suggested to them upon calling the STI clinic for a physical appointment.

#### Uptake of Home-Based Testing

**Fig. 2:**
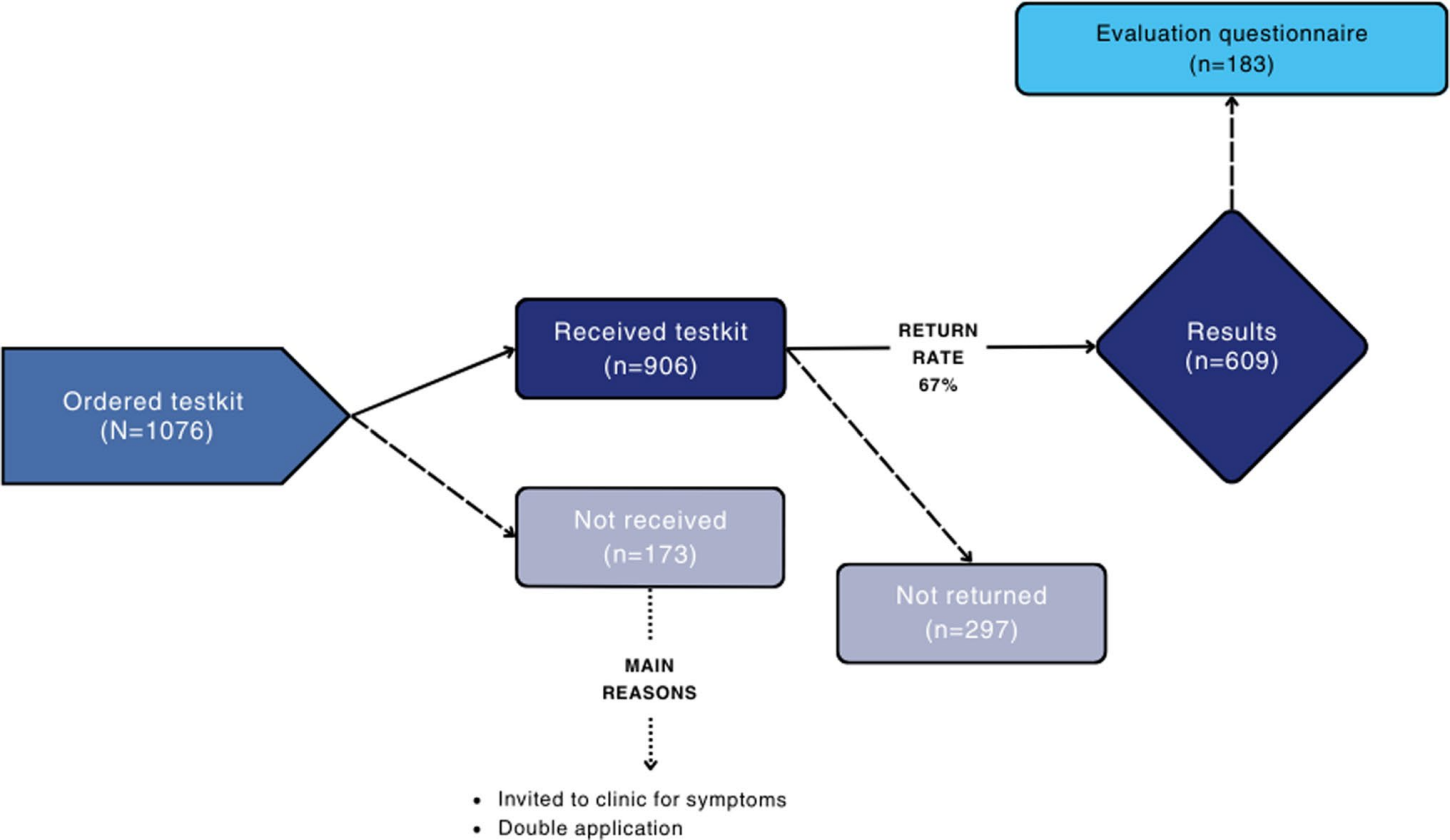
Flowchart from order to evaluation of home-based sexual health care in Limburg4zero

Figure 2 shows the participants’ flowchart from order to evaluation. From April 2021 to January 2024, 1076 test kits were requested and 906 (84%) of them were sent to participants. The main reasons for not sending test kits were the choice of the HCP to invite participants to the clinic because of specific symptoms, a double request, or repeated requests within six months after the last test (without valid reason). Of the test kits that were sent to participants, 67.2% (609) returned and all participants received their results successfully.

#### Demographic Variables

Of participants who received home-based sexual health care (n = 906), 64% had sex only with men in the past six months (579/906), and the median age was 31 years (IQR 26–38). Most participants had Western ethnicity 92.7% (840/906) and a college or university degree (57.7%; 523/906), more than half of participants lived in non-urbanized areas 55.0% (498/906). The median number of sex partners in the past six months per participant was 3 (IQR 2–4). CAI with a casual partner was indicated by 47.2% (387/906) and 13.9% (126/906) engaged in chemsex in the past 6 months.

#### Returned vs. not Returned

Table 2 shows the characteristics of participants who returned their self-sampling test kit, and participants who did not return their test kit. Among participants who returned their self-sampling test kit, most had Western ethnicity (94.2%; 551/585), university or college degree (65.1%; 355/585), and 53.8% (314/585) lived in an urbanized area. About four in every ten participants (41.2%; 241/585) indicated having CAI in the last 6 months and 37.1% (217/585) had never tested for HIV before. Participants who did not return their self-sampling test kit had a Western ethnicity and indicated chemsex more often (p < 0.05).

**Table 2:** Characteristics of participants that returned or did not return the STI/HIV self- sampling test kit

Characteristics	Returned test kit (n = 609)	Not returned test kit (n = 297)	*p*
	% of total (n)	% of total (n)	
Ethnicity^a^			**0.011**
Western	94.3 (574)	89.6 (266)	
Non-western	5.7 (35)	10.4 (31)	
Age^2,d^			0.062
< 25 yr	26.0 (158)	31.1 (91)	
26–37 yr	37.0 (225)	39.6 (116)	
> 37 yr	37.0 (225)	29.4 (86)	
Level of urbanization^d^			0.449
Urbanized	45.7 (278)	43.1 (127)	
Non-urbanized	54.3 (330)	56.9 (168)	
Education^d^			0.125
College or university	60.9 (366)	54.0 (157)	
Vocational training	29.5 (177)	35.7 (104)	
Less than high school	9.7 (58)	10.3 (30)	
Gender of sexual partners^d^			**0.039**
Only men	66.8 (405)	59.8 (174)	
Both men and women	33.2 (201)	40.2 (117)	
Number of sexual partners^b,c^			0.885
0–2	35.5 (216)	37.0 (110)	
2–4	33.7 (205)	33.3 (99)	
> 4	30.9 (188)	29.6 (88)	
CAI^c^			0.883
Yes	66.2 (403)	66.7 (198)	
No	33.8 (206)	33.3 (99)	
Chemsex			**0.005**
Yes	11.7 (71)	18.5 (55)	
No	88.3 (538)	81.5 (242)	
Ever tested HIV			**0.018**
Yes	63.4 (386)	55.2 (164)	
No	36.6 (223)	44.8 (133)	

^a^Ethnicity and level of urbanization were based on definitions used by the Central Bureau of Statistics (NL) (http://www.cbs.nl)

^b^Age groups and number of sexual partners were based on tertile distributions

^c^In the past 6 months

^d^Characteristics did not count to 100% due to: 0.6% missing data in age, 0.3% missing urbanization data, 1.5% uncategorizable educational level, 1.0% incongruence in data regarding gender of sexual partner

### Effectiveness

Table 3 shows the sexual (testing) behavior and sociodemographic characteristics of participants who received a self-sampling test kit, compared to MSM who attended the clinic for STI and HIV screening in Limburg. Of all test kit participants, 39.3% (356/906) never tested for HIV before, 38.8% (352/906) did not test regularly (every 6 months), compared to 28.1% of clinic attendees who never tested for HIV before (1225/4356), (p < 0.001). The majority, 62.5% of test kit participants (566/906), had not tested for any other STI in the past twelve months. In comparison to test kit participants, clinic attendees were more likely to have had 4 or more sex partners (30.5% vs. 43.7%; p < 0.05). There were fewer test kit participants with less than high school education (9.7%; 88/906) and non-western ethnicity (7.3%, 66/906) compared to clinic attendees (p < 0.05).

**Table 3:** Characteristics of participants that received a home-based test kit compared to clinic attendees (MSM without HIV, that did not use PrEP from April 2021–December 2024)

Characteristics	Received home- based test kit (n = 906)	Clinic attendees (n = 4356)	*p*
	% of total (n)	% of total (n)	
Ethnicity^a^			**0.000**
Western	92.7 (840)	87.6 (3818)	
Non-western	7.3 (66)	12.3 (537)	
Age^b,&^			0.078
< 25 yr	27.5 (249)	24.7 (1078)	
26–37 yr	37.6 (341)	37.2 (1619)	
> 37 yr	34.3 (311)	38.1 (1659)	
Level of urbanization^a,d^			0.302
Urbanized	44.7 (405)	45.2 (1970)	
Non-urbanized	55.0 (498)	51.5 (2245)	
Education^d^			**0.000**
College or university	57.7 (523)	49.3 (2149)	
Vocational training	31.0 (281)	30.2 (1314)	
Less than high school	9.7 (88)	15.1 (656)	
Gender of sexual partners^d^			0.05
Only men	63.9 (579)	67.6 (2946)	
Both men and women^e^	35.1 (318)	32.0 (1396)	
Number of sexual partners^b,c^			**0.000**
0–2	36.0 (326)	30.6 (1135)	
2–4	33.6 (304)	25.6 (1116)	
> 4	30.5 (276)	43.7 (1905)	
CAI^c^			0.232
Yes	66.3 (601)	65.2 (2842)	
No	33.7 (305)	34.4 (1497)	
Chemsex			**0.031**
Yes	13.9 (126)	16.8 (732)	
No	86.1 (780)	83.2 (3624)	
Ever tested HIV			**0.000**
Yes	60.7 (550)	71.9 (3131)	
No	39.3 (356)	28.1 (1225)	

^a^Ethnicity and level of urbanization were based on definitions used by the Central Bureau of Statistics (NL) (http://www.cbs.nl)

^b^Age groups and number of sexual partners were based on tertile distributions

^c^In the past 6 months

^d^Characteristics did not count to 100% due to: 0.6% missing data in age, 0.3% missing urbanization data, 1.5% uncategorizable educational level, 1.0% incongruence in data regarding gender of sexual partner

^e^Since 2023, gender has been extended with other gender identities. Sex with other gender identities is characterized as sex with both men and women

#### Previously Untested Subgroups

Table 4 shows the different characteristics between previously tested and untested participants. There were significant differences between tested and untested subgroups in terms of age, level of education, gender of sexual partners, and the number of sexual partners. There were more participants younger than 25 years who never have tested for HIV before, than among previously tested participants (35.7% vs. 22.4%, p < 0.05). Among previously tested participants, the proportion with a college or university degree was higher than among those who had never been tested (63.5% vs. 51.1%, p < 0.05). The proportion of participants that have tested previously, were more often men that only have sex with men (50.1%, p < 0.05), and participants with more than 4 sexual partners (37.5%; p < 0.05).

**Table 4:** Characteristics participants who received an STI/HIV self-sampling test kit and previously tested for HIV versus previously untested participants

Characteristics	Previously tested HIV (n = 550)	Previously untested HIV (n = 356)	*p*
	% of total (n)	% of total (n)	
Ethnicity^a^			0.303
Western	92 (506)	93.8 (334)	
Non-western	8 (44)	6.2 (22)	
Age^b,d^			**0.000**
< 25 year	22.4 (123)	35.7 (126)	
26–37 year	41.6 (228)	32.0 (113)	
> 37 year	35.9 (197)	32.3 (114)	
Level of urbanization^d^			0.070
Urbanized	47.3 (259)	41.1 (146)	
Non-urbanized	52.7 (289)	58.9 (209)	
Education^d^			**0.001**
College or university	63.5 (344)	51.1 (179)	
Vocational training	27.3 (148)	38.0 (133)	
Less than high school	9.2 (50)	10.9 (38)	
Gender of sexual partners^d^			**.000**
Only men	73.9 (405)	49.9 (174)	
Both men and women	26.1 (143)	50.1 (175)	
Number of sexual partners^b,c^			**0.000**
0–2	29.1 (160)	46.6 (166)	
2–4	33.5 (184)	33.7 (120)	
> 4	37.5 (206)	19.7 (70)	
CAI^c^			0.376
Yes	67.5 (371)	64.6 (230)	
No	32.5 (179)	35.4 (126)	
Chemsex			0.377
Yes	13.1 (72)	15.2 (54)	
No	86.9 (478)	84.8 (302)	

^a^Ethnicity and level of urbanization were based on definitions used by the Central Bureau of Statistics (NL) (http://www.cbs.nl)

^b^Age groups and number of sexual partners were based on tertile distributions

^c^In the past 6 months

^d^Characteristics did not count to 100% due to: 0.6% missing data in age, 0.3% missing urbanization data, 1.5% uncategorizable educational level, 1.0% incongruence in data regarding gender of sexual partner

#### Sexual Health Information

Depending on the given answers, tailored sexual health information was available to all participants. We informed participants who never used PrEP before (96.4%) about the possibilities of PrEP and participants who were not vaccinated for Hepatitis B (41.6%) about free-of-charge Hepatitis B vaccinations. Participants who reported drug or alcohol use before or during sex and lived in a certain postal code area (26.8%) were informed about a regional chemsex consultation initiative. Only 12.5% of participants used the possibility of a phone call with an HCP for additional counseling (i.e., counseling is standard of care when visiting an STI clinic).

#### New Diagnoses

CT was found in 6.1% (37/609), and 7.9% (48/609) MSM were diagnosed with NG. A few 1.8% (11/609) new syphilis infections were found and a single new HIV diagnosis (0.2%; 1/609). HCP invited 27.4% (167/609) participants to the clinic for confirmatory testing and care after a reactive test result 20.1% (122/609) or invalid blood samples 7.4% (45/609). Among MSM (without HIV, not PrEP using) who attended an STI clinic, 0.6% (26/4356) were newly diagnosed HIV, 9.6% (420/4356) CT, 10.5% (456/4356) NG, and among 1.6% (70/4356) syphilis was diagnosed.

#### Acceptability Among MSM

An evaluation questionnaire was issued to assess acceptability towards home-based sexual health care after participants received their test results. A total of 183 (31.2%) participants completed this questionnaire. On a scale from 0 to 100, performing the finger-prick blood collection was assessed as the most difficult (71.2), and the collection of urine was assessed as the least difficult (15.6). Two-thirds of participants (66.7%; 122/183) indicated they would recommend the home-based test kit to others. Participants reviewed the test kit with an average of 8.6 out of 10. Most of the participants would use the home-based test kit again in the future (83.6%; 153/183). Open-ended responses in the questionnaire emphasized the high acceptability of the test kit, specifically the convenience and privacy of performing tests at home. Participants described the tests as ‘easy’, ‘clear’, and ‘timesaving’. Participants also highlighted practical difficulties with collecting blood via finger-prick such as not being able to get enough blood, spilling blood, or experiencing unease when performing the prick. Additional data from in-depth interviews with MSM confirmed these findings, the benefits of a previous clinic visit were indicated: *“With those good instructions and if you have been to the GGD [STI clinic] yourself before, then it just seems easier to me, because then you also do it yourself. So-”* (MSM, interview #2; on modeling). In-depth interpretation of difficulty in blood collection was: *“(…) it really took me a few minutes before I even got the first drop into the er, into the er, bottle, well, whatever. And then I spent quite a long time fidgeting to get more blood in. So that was the only thing I was thinking like, maybe there’s an easier way to do that.”* (MSM, interview #3; on performing blood test)*.* Members of the CAB emphasized home-based sexual health care complementary to clinic-based sexual health care. They confirmed that important barriers to accessing clinic-based care were expected fear and shame to face an HCP or others they may encounter at the clinic, lack of knowledge about sexual health, expected costs, language barriers, and distrust in government institutes.

### Adoption

#### Determinants Associated with the Innovation

Although HCPs perceived the urine and swab tests as easy, because they already ask clients to swab themselves in the clinic, some HCPs expressed worries about the correctness and complexity of blood sampling: *“And certainly a blood test, those other tests are not such a world disaster, but apparently taking blood is really a thing, isn’t it?”* (STI clinic nurse, interview #3, on sampling concerns). In addition, a few HCPs were concerned about the correctness of syphilis diagnostics with self-sampled blood. Some HCPs described challenges with accessibility for practically educated men: *“But just those with lower economic positions are little self-reliance in this. They are not going to read a piece of text, then you have already lost them.”* (STI clinic nurse, interview #3, on complexity for low educated). Images and video instructions were recommended to enhance the clarity of instructions.

#### Determinants Associated with the Implementer

STI clinic HCP’s role in home-based testing consisted specifically of handling the logistic process of sending and receiving self-sampling tests and managing the clients who need follow-up consultation (i.e., treatment as regular care). The role of other HCPs, such as GPs, was limited to creating awareness of the availability of home-based testing among their patients. All HCPs had a positive attitude towards implementing health care innovations such as home-based sexual health care. They expected low complexity for themselves to adopt and promote the intervention and indicated high self-efficacy. Although some HCPs expected colleagues to have concerns, most of them expected colleagues to be positive about working with home-based initiatives (social norm). STI clinic HCP highlighted previous experience with innovations, which may affect outcome expectations: “*My only concern is that it will cause another fuss like it did with syphilis (note: a previous project with syphilis testing at home). That you don’t have enough information from the clients, that something comes up that you don’t know about yet. We’ll have to see that again.”* (STI clinic Nurse, interview #3, on previous experience)*.* The main described challenge of home-based sexual health care is the lack of (non-verbal) communication with the client thereby lacking relevant information and the inability to educate clients. *“But also, suppose here in the face-to-face conversation you always try to respond to non-verbal signals. See if you can still figure things out. Ehm, of course, you miss that.”* (STI clinic nurse, interview #1, on client communication). Most of the GPs indicated that knowledge about (home-based) sexual health care outside the STI clinic may be inadequate: *“Eh, also in terms of knowledge, I think. I think that the GGD [STI clinic] naturally has more knowledge than the average, well, average GP. (…) Because you come into contact with it more, so you can also communicate it better, I think.”* (GP, interview #13, on knowledge gap). STI clinic HCP examined the implementation as compatible with their current work duties. GPs also described their professional obligation in the home-based testing as mostly informative, as they assign responsibility for these tests to STI clinic HCP.

#### Determinants Associated with the Organization

Expected organizational benefits were related to increased work efficiency, such as offering a wider range of care, consultations focused on more vulnerable populations and less often having to deal with no-shows from clients. Another benefit would be task shifting: *“Yes, so, also a bit of efficiency of course. (…) you’re going to have a bit of a task shifting, which will create more space in other disciplines. So that in itself is, an advantage of, this kind of, innovation.”* (STI clinic doctor, interview #14, on time and cost-efficiency). A few HCPs emphasized the relevance of testing outside the clinic, also regarding external context factors, such as restrictions and down-scaling of clinic-based care during the COVID-19 pandemic. One HCP described the sudden amplification of home-based opportunities due to the global pandemic: *“Then I think that is a very good development, and I think we have made tremendous progress last year with corona because we had to start providing care remotely at once. Ehm, so yes, you know, I think it is a solution for a, for a very large target group, isn’t it?*” (GP, interview #10, on remote care). Most of the HCPs highlighted the importance of the involvement of the team in the implementation, yet being informed via a presentation or training would be enough. Among GPs, a transparent way of communicating is preferred to enhance the collaboration between them and the STI clinic: “*Well, (…) I would like it if you just received a message saying, your patient has been to us, and this is what we did. And that I don’t do the same thing again the same week, when that is not necessary, or *vice versa*. I think that in itself is also a very, um, nice, nice way of working together-* “(GP, interview #9, on communication between STI clinic and GP). In addition, some GPs indicated platforms via which this communication can take place (e.g., existing digital platforms, e-mail or call with direct contact). Some GPs mentioned that they value the training and support from the STI clinic and prefer additional training sessions to gather more information on sexual health in general.

### Implementation

#### Fidelity

Table 5 examined different key elements and their implementation fidelity. Key elements were drawn from the implementation script and categorized into ‘Intervention’, ‘Research’, and ‘Communication’. Nearly all key elements were implemented. The planning group monitored the implementation and had regular meetings where these elements were discussed, and changes were decided upon. A chair and structural meetings were required to maintain organization and adherence to tasks. Most of the implementation elements needed adjustments. A high degree of flexibility was required from HCP and efficient communication to adapt the intervention without changing key elements. Although the planning group intended to create an online platform for HCP, this element could not be implemented. This was mainly due to cost restrictions and dependence on external parties, often beyond the control of the implementers. Without an online platform, several actions needed manual action, which was time-consuming. All adjustments to the implementation were to reduce the workload of HCP. For example, we added video instructions for enhanced clarity of the testing process and performance of blood sampling, to ensure increased returned test kits with sufficient blood samples. Therefore, HCPs needed to follow up less on sufficient blood samples from clients (i.e., by inviting them to the clinic). In addition, we established a website (http://www.limburg4zero.nl) with all the details of home-based sexual health care, including videos and other informative content. In the research category, both elements of monitoring and evaluation research were implemented without adjustments. The communication strategy was implemented in phases that matched the implementation phases.

**Table 5:** Overview to what extent key elements are implemented in practice

Category	Key elements	Implemented?	Adjustments?
Intervention	Content of test kit	Yes	Yes, addition of an instructional video
	Website limburg4zero	Yes	Yes, added information
	Online anamneses development	Yes	Yes, added questions
	Online platform for HCP	No	–
	Daily registration	Yes	Yes, different role division
	Sending process test kits	Yes	Yes, added take away option
	Returning process test kits	Yes	Yes, the algorithm of blood sample testing
	Client treatment	Yes	No
Research	Process monitoring	Yes	No
	Evaluation research	Yes	No
Communication	Realization of communication strategy	Yes	Yes, added channels

#### Maintenance

Currently, the implementation of home-based sexual health care (Limburg4zero) is in its final phase, where this service will be embedded in regular sexual health care. Therefore, we provided recommendations to increase the maintenance of home-based sexual health care for the priority population and HCP.

#### Priority Population Recommendations

Future optimization of the service may be in remote consultations. Of all participants, 37.9% (408/1076) would be interested in remote consultations, preferably through telephone (53.9%) or video call (29.9%). The preferred topics for this consultation were PrEP use (46.6%; 190/408), testing possibilities (34.3%; 140/408), and dealing with positive STI test results (30.4%; 80/408). In the evaluation questionnaire, open responses provided recommendations for maintenance of the home-based sexual health care in general. Most participants would prefer home-based sexual health care to be available for more populations, such as MSM who use PrEP. Some suggestions emphasized the development of the test kit itself, such as more inclusive language. Consequently, more individuals may be able to use the test kit. However, further research is necessary to determine the needs of clients with less than a high school education. In addition, members of the CAB proposed online guidance options for first-time testers. More than half of participants (55.2%; 101/183) preferred home-based sexual health care complementary to clinic-based sexual health care. Therefore, both home-based and clinic-based options should be made available to individuals seeking sexual health care.

#### HCP Recommendations

There was a high willingness among STI clinic HCPs to continue engaging in home-based sexual health care in the future. Members of the planning group have the aspiration to expand the service to other subgroups or to make it available at more locations (e.g., outreach, other regions). For HCPs -outside of the planning group- to use the innovation, certain adaptations on innovation and organizational level need to be considered. Most HCPs indicated the importance of a comprehensive and tailored offer, which clients may choose from: *“Ehm, maybe in the future you say, I’ll do a home test once, then I’ll come to the GGD [STI clinic], then I’ll do another home test, I’ll come to the GGD again.”* (STI clinic nurse, interview #1, on future perspectives). However, some conditions were assessed. First, there is a considerable need for an online platform for HCP that is designed for home-based options. HCP indicated that it is not desirable to keep performing manual actions in the long term. Additionally, current manual actions for HCP and laboratory may restrain further integration of home-based sexual health care (e.g., for more populations). Second, STI clinics and other adopting organizations must be prepared to invest time, costs, and personnel in the implementation of home-based sexual health care. The formation of a planning group is crucial to lead the implementation process. Especially for maintenance in the long term and integration into regular sexual health care.

## Discussion

This study used PRISM to systematically evaluate the implementation and outcomes of home-based sexual health care among MSM living in Limburg, the Netherlands. Participants received home-based sexual health care, including self-sampling STI/HIV testing, and tailored sexual health information. Home-based sexual health care participants have more often never been tested for HIV before, compared to clinic attendees. The overall acceptability of home-based sexual health care was high among MSM and HCPs (implementers). Key elements of home-based sexual health care were mostly implemented as designed. For maintaining the implementation, some individual and implementer adaptations may be considered to guarantee sustainability in the long term.

### Previously Untested MSM

In the Netherlands, we were approaching zero new HIV diagnoses in 2023. However, this last mile demands sincere effort to end the HIV epidemic [3]. Tailoring different methods of communication may increase reaching populations that are at elevated risk for HIV but remain untested. A previous study from the United Kingdom among black African individuals examined that self-sampling interventions should contain technology that enhances autonomy and should be focused on the populations, acknowledging privacy and stigma concerns [25]. A study among MSM in the UK showed that young men prefer more traditional settings for receiving their sexual health information (i.e., HCP, GP, hospital) [26]. This aligns with findings from this study, where most MSM were reached via the STI clinic HCP. Considering these outcomes, a combined strategy may be a recommended solution for reaching untested individuals. Generally, return rates of self-sampling test kits ranged between 57% and over 80%, corresponding with the 67% return rate in this study [27–31]. Reasons for not returning samples were concerns about a positive result, confidentiality, the timespan for receiving results, and the finger prick for a blood sample [32]. Recommendations for maintaining or increasing the return rates were to provide incentives for the return of the test kit [30]. Messages specifically designed to target behavior by evoking reciprocity and using prompts increased the return rate of home-based test kits [33].

A key finding in this study is the high number of participants who have never tested for HIV before. A previous study from China among MSM showed a similar number of first-time testers (42.9%) [34]. Other studies emphasize the preference for home-based testing by never-testing MSM [7, 9]. Although we might assume that testing previously untested MSM may uncover new HIV diagnoses, only a single new HIV was confirmed. In other studies, similar small positivity rates (< 1%) among home-based test kit users were found [35–37], some even without any new cases [38–40]. However, a recent Spanish study on self-sampling testing demonstrated a high HIV reactivity rate (2.8%) among MSM and transwomen [31]. Previous research showed the association between a lower (than high school) educational level and more difficulties in performing HIV self-test [41]. Results in this study also confirmed inadequate participation among lower-than-high-school-educated and migrant MSM. In its current form, home-based sexual health care may not be accessible for these subgroups. Therefore, instructions should be adapted to their needs, with the use of supporting materials (e.g., pictograms, audio instructions, supervision). A pilot evaluation among American MSM advised additional video material and images of the puncture site to benefit the correct performance of HIV self(sampling) testing [42, 43]. In addition, tailoring the intervention to migrant subgroups may require translation into local and culture-sensitive languages [44]. Further, participants of the test kit should be linked to additional sexual health care information such as STI prevention measures (i.e., PrEP, safe sex) [45, 46]. E-health platforms or apps may serve as a tool to easily deliver additional sexual health care advice or information [47].

### Health Care Providers

Previous pilot evaluations indicated a high adoption rate for home-based sexual health care among HCPs [48]. For HCPs, the most important motivation to adopt home-based testing was the expected benefits for their MSM clients such as increased accessibility, anonymity, and convenience. Adoption concerns may be characterized as concerns about follow-up, missing face-to-face contact, and eventually inadequate engagement with regular sexual health care (in the clinic) [49]. Challenges could be summarized in the inability to provide quality care to clients. Previous research among Canadian service providers also showed an accelerated implementation of new and innovative sexual health services due to the global COVID-19 pandemic [50]. As a consequence, service providers might have experienced that home-based sexual health care may be a convenient, accessible, and safe alternative to regular clinic-based testing. However, considering widely accessible sexual health care, home-based sexual health care should be implemented complementary to clinic-based health care [30]. It is crucial to assess the needs of the priority population and implementers, to determine which strategy could reach untested subgroups while maintaining quality sexual health care [51]. In addition, research from an implementers’ point of view was limited, resulting in an underrepresentation of their needs in home-based sexual health care. In terms of implementation fidelity, most key elements in this study were implemented as designed in the implementation script. The implementation was characterized by a combined approach of research elements and sexual health care practice. Implementers were involved in the early development phase to tailor needs, share knowledge, create trust, and work towards a shared goal [52]. The multidisciplinary collaboration was stable and reflected in the structure of the planning group, collaboration with external organizations in the region, and task distribution. However, challenges in the automation of the test kit ordering process required implementers (HCP, laboratory) to perform time-consuming manual actions. Therefore, a high degree of flexibility is necessary to maintain implementation fidelity, without compromising key elements of the intervention [53].

### Future Implications

Insights of this study may offer implications for future implementation of complex interventions such as home-based sexual health care. Developing an evidence-based intervention is crucial. Previous research has identified IM as the most comprehensive approach to maximize the success of interventions [54]. However, when implementing such an innovation, employing a systematic implementation strategy can enhance the likelihood of successful outcomes. This process may involve determining the priority audience, selecting appropriate materials, and outlining the steps needed to establish an effective sexual health care service. A theoretical framework, such as PRISM, can provide valuable support in this endeavor [18]. Further, educating other implementers (HCP) and raising awareness about prevention strategies was a recurring theme and could be addressed in different educational activities and online e-learning programs [55, 56]. Additionally, efforts to improve return rates of test kits should be made by examining the reasons for non-return. To accomplish future implications, structural support (i.e., financial, workload) from organizations may be needed to enhance a sustainable implementation process. For example, this may include a planning group that can take the lead in implementation and financial support to equip tools that enhance the implementation process.

### Strengths and Limitations

This study examined the implementation of the first STI clinic distributed comprehensive home-based sexual health care for MSM in the Netherlands. A methodological strength of this study is the use of a systematic framework (PRISM) informed by multiple theories and findings [18]. One of its achievements is the high number of reaching previously untested MSM with sexual health care. Our study should also be viewed in light of some limitations. First, MSM who participated in this study had a university or college degree and were mostly of Western ethnicity than MSM who attended a clinic (Table 3). This might have led to bias in the perspectives of these MSM subgroups on home-based sexual health care. Therefore, needs assessments should be conducted among more vulnerable populations to determine if home-based testing might be fitting or if these groups should be prioritized for clinic-based sexual health care. Second, it was not possible to determine the cost-effectiveness of this complex approach. Although we briefly investigated the time that the implementers took to process an order, we were not able to calculate the efficiency in terms of financial cost. Future research may assess the cost-effectiveness of a home-based sexual health care service. Third, due to data constraints it was not possible to examine follow-up consultations upon given sexual health information. Although HCPs informally confirmed some clients coming in for PrEP counseling or Hepatitis B vaccination who have visited the STI clinic, we could not determine the effectiveness of the sexual health information. Lastly, at this stage, we only diagnosed a few new STI and HIV among home-based sexual health care participants. Since we reached 39.3% previously untested MSM with home-based testing, we might have expected the detection of more new diagnoses.

## Conclusion

The implementation of home-based sexual health care is a continuous, cyclic process of careful monitoring and optimization, ideally within a systematic framework. Results to date indicate high acceptability among MSM and HCP, successfully reaching MSM who had never previously tested for HIV. Further optimization and adjustments are anticipated to enhance inclusivity for vulnerable groups and ensure the long-term sustainability of home-based testing services. The proposed amendments to the procedure may further amplify the positive effects of home-based sexual health care. Therefore, adopting a home-based sexual health care approach is a necessary complement to clinic-based sexual health care.

## Supplementary Information

Below is the link to the electronic supplementary material.

Supplementary file1 (PDF 192 kb)—Pathway of home-based sexual health care in Limburg4zero

Supplementary file2 (PDF 588 kb)—Questionnaire (anamneses) when ordering self-sampling STI/HIV test kit

Supplementary file3 (PDF 372 kb)—Evaluation questionnaire Limburg4zero

Supplementary file4 (PDF 198 kb)—Communication material for promoting Limburg4zero among MSM in the Limburg region of the Netherlands

Supplementary file5 (PDF 154 kb)—Characteristics participants semi-structured interviews with HCP (n=14)

## Data Availability

The data of this study contain potentially identifying and sensitive participant information. Due to the General Data Protection Regulation, it is not allowed to distribute or share any personal data that can be traced back (direct or indirect) to an individual without consent (or based on another legal basis). Publicly sharing the data would not be in accordance with the participants’ consent obtained for this study. Therefore, data used and/or analyzed during the study are available from the head of the data archiving of the Public Health Service South Limburg on reasonable request. Interested researchers should contact the head of the data-archiving of the Public Health Service South Limburg (Helen Sijstermans: helen.sijstermans@ggdzl.nl) when they would like to re-use data.
