# Supplementary figures and images for: Evaluating the Implementation of Home-Based Sexual Health Care Among Men Who Have Sex with Men: Limburg4zero
Source: AIDS Behav. 2025 Jan 8;29(3):976–92. doi: 10.1007/s10461-024-04579-6 (PMC11830641; doi:10.1007/s10461-024-04579-6)

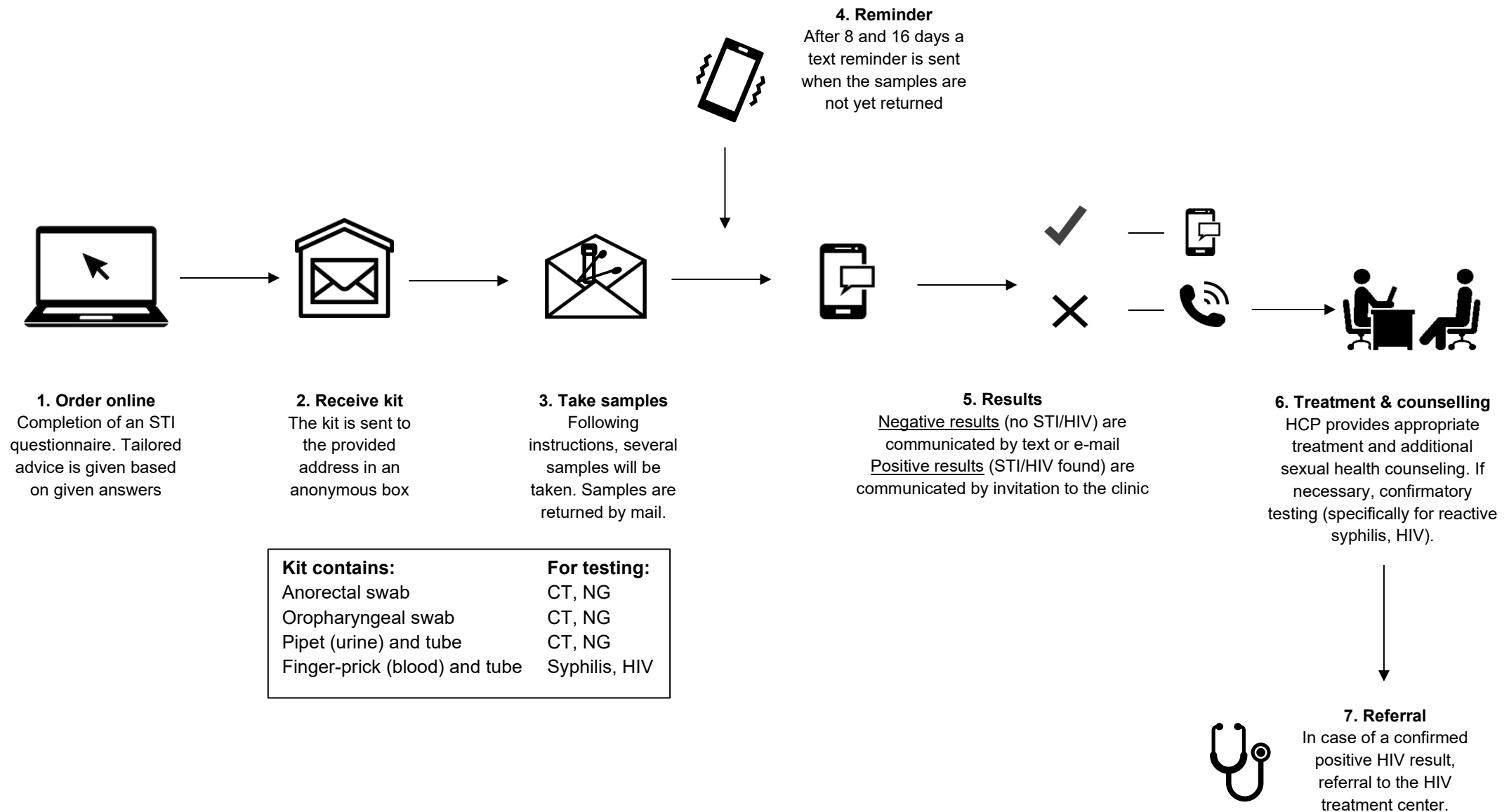

Supplement: Supplementary file 1 — Supplementary file1 (PDF 192 kb)—Pathway of home-based sexual health care in Limburg4zero [file 10461_2024_4579_MOESM1_ESM.pdf]
