# Supplementary material for: Evaluating the Implementation of Home-Based Sexual Health Care Among Men Who Have Sex with Men: Limburg4zero
Source: AIDS Behav. 2025 Jan 8;29(3):976–92. doi: 10.1007/s10461-024-04579-6 (PMC11830641; doi:10.1007/s10461-024-04579-6)
Supplement: Supplementary file 2 — Supplementary file2 (PDF 588 kb)—Questionnaire (anamneses) when ordering self-sampling STI/HIV test kit [file 10461_2024_4579_MOESM2_ESM.pdf]

Supplementary material S2. Questionnaire (anamneses) when ordering self-sampling STI/HIV test kit

Are you a man who also has sex with men? Do you live in the province of Limburg (NL)? Then you can apply for our self-sampling test kit!

Fill out the online application below to get a free reliable and discrete self-sampling testkit. These tests are offered to you by the STI clinic of the Center for Sexual Health (Public Health Service; GGD) in Limburg and allows you to check for sexually transmitted infections such as chlamydia, gonorrhea, syphilis and HIV. After you have received the tests from us, you collect the samples yourself and return them for your results. The instructions in package will tell you step by step how to collect and send in the tests.

The self-sampling test kit is part of Public Health Service (GGD) home-based sexual health care. Hence, we ask you to completely fill in your application. The application is part of the Public Health Service (GGD) care and therefore public policy rules of the Center for Sexual Health apply.

1. How did you find www.limburg4zero.nl to order the self-sampling testkit? You can give one or more answers
    a. Via a card that I have seen/been given by a healthcare provider (e.g., GGD, GP, pharmacy)
    b. Via a card I have seen/been given by someone I know (e.g., friend, acquaintance)
    c. Via a card that I saw/got at a bar or entertainment venue
    d. Via a card that I saw/got at a sauna
    e. Via a card I saw/got at a cruising area
    f. Via a card I saw/got at another public location (e.g., supermarket, bus stop, sex shop)
    g. Via a screen and/or poster that I have seen at a health care provider (e.g., GGD, GP, pharmacy)
    h. Via a screen and/or poster that I have seen in a bar or entertainment venue
    i. Via a screen and/or poster that I‘ve seen at a sauna
    j. Via a screen and/or poster I‘ve seen at a cruising area
    k. Via a screen and/or poster that I’ve seen at another public place (e.g., supermarket, bus stop, sex shop)
    l. Online, through a post on social media (e.g., Facebook, Instagram, LinkedIn)
    m. Online, via a banner or advertisement (e.g., Grindr)
    n. Online, via another website
    o. Heard about it from an acquaintance
    p. Heard about it from a health care provider
    q. Read about it in a regional newspaper (e.g., De Limburger, WijLimburg)
    r. Seen on regional television (e.g., L1 TV)
    s. Heard on regional radio (e.g., L1 radio)
    t. Other, please specify…
    u. I would rather not say

2. What is your postal code?
    a. Enter the 4 digits of your postal code

3. What is your gender?
    a. Man
    b. Woman
    c. Transgender

4. Indicate which statement applies to you:
    a. I feel like a man with female genitalia.
    b. I feel like a man with a neo-penis.
    c. I feel like a woman with a neo-vagina.
    d. I feel like a woman with male genitalia.
    e. I feel neither man nor woman; I was born a man with a penis.
    f. I feel neither man nor woman. I was born a man with a vagina.
    g. I feel neither man nor woman. I was born a woman with a vagina.
    h. I feel neither man nor woman; I was born a woman with a penis.

5. Do you have sex with men?
    a. Yes
    b. No

6. Have you ever had sex with men in the past?
    a. Yes
    b. No

7. Have you ever been tested for HIV?
    a. Yes
    b. No

8. Did you receive a positive result to this HIV test?
    a. Yes, I am HIV positive
    b. No

9. Have you used PrEP in the past year?
    a. Yes, in the past 3 months
    b. Yes, 4 - 12 months ago
    c. No

10. Who is/was the prescriber of PrEP?
    a. GGD
    b. GP
    c. HIV treatment specialist
    d. The city clinic
    e. Another doctor/specialist
    f. PrEP study (AMPREP, INPREP, Discover)
    g. Acquired through friends or sex partners
    h. Acquired or purchased abroad
    i. Purchased online
    j. Other, please specify…

11. How did you use PrEP?
    a. Daily
    b. Around sexual activity (always)
    c. Around sexual activity (I only use PrEP, whenever I think I am at risk of contracting HIV)

12. Reasons for stopping PrEP:
    a. I experienced side effects
    b. I have had no or low risk of contracting HIV

c. Logistical reasons
d. Renal impairment
e. HIV positive
f. I forgot to take the PrEP more often
g. Unknown
h. Other, please specify…

13. Have you ever had syphilis?
    a. Yes
    b. No

14. Do you have any symptoms? *Symptoms such as warts, blisters, rashes, fever or sore throat*
    a. Yes
    b. No

15. Indicate your symptoms:
    a. Secretion from the urethra
    b. Anal discharge
    c. Altered vaginal discharge active condition
    d. Warts
    e. Pain/burnt feeling when urinating
    f. Anal blood loss
    g. Blood in urine
    h. Vaginal bleeding during/after sex active condition
    i. Vaginal bleeding not caused by sex active condition
    j. Pain in the scrotum
    k. Painful sores/bladders on the genitals
    l. Painful wounds/bladders on the anus
    m. Painless wounds/bladders
    n. Pain in anus during/after sex
    o. Pain in genitals during/after sex
    p. Rash
    q. Itching of genitals/anus
    r. Sore throat (without a cold)
    s. Fever
    t. Fever or flu-like symptoms without a cold
    u. Swollen lymph nodes
    v. Other, please specify…

16. Have you been warned for an STI?
    a. Yes
    b. No

17. What STI were you warned for?
    a. Chlamydia
    b. Hepatitis B
    c. LGV
    d. Hepatitis C
    e. Gonorrhea
    f. HIV
    g. Syphilis/lues
    h. STI unknown

i. Other, please specify…

18. I have been warned via...
    a. Partnerwaarschuwing.nl
    b. Personally
    c. Notification or letter
    d. Health care provider (e.g., doctor, nurse, GGD)

19. I have been warned by...
    a. Anonymous/Unknown
    b. Sex partner from less than 2 months ago
    c. Sex partner from more than 2 months ago

20. When were you warned for contracting HIV?
    a. Less than 72 hours ago
    b. More than 72 hours ago

21. Are you currently attending education?
    a. Yes
    b. No

22. What is your level of education?
    a. No education
    b. Elementary school
    c. Pre-vocational education (Lbo/Mavo/VMBO)
    d. Middle level applied education - level 1 (MBO niveau 1)
    e. Middle level applied education - level 2 through 4 (MBO niveau 2-4)
    f. Preparatory higher general education (Havo/VWO/Gymnasium)
    g. Higher education (HBO/WO, University, Post-academic)
    h. Other, please specify…

23. Which field of study are you attending?

24. Are you on any medication?
    a. Yes
    b. No

    Name medication:

25. Are you allergic to any medication?
    a. Yes
    b. No

    Name medication:

26. Have you taken antibiotics in the last month?
    a. Yes
    b. No

    Name antibiotics: (also note start and stop date)

27. Are you allergic to antibiotics?
    a. Yes
    b. No
    c. I do not know

Name antibiotics:

28. Did you use drugs or alcohol before/during sex?
    a. Yes, in the last 6 months
    b. Yes, more than 6 months ago
    c. No

29. What substances did you use before/during sex?
    a. Alcohol: tipsy/drunk
    b. Poppers
    c. Heroin
    d. XTC/MDMA
    e. Weed/hash
    f. Cocaine
    g. Speed
    h. Ketamine
    i. GHB/GBL (“Gina”)
    j. Crystal Meth (“Tina”)
    k. Mephedrone (“Miau Miau”)
    l. 3-MMC
    m. 4-MEC
    n. 4-FA
    o. Erectile stimulants
    p. Other, please specify…

30. Do you use IV needles/inject drugs/slamming?
    a. Yes
    b. No

31. Have you taken an STI test in the last year?
    a. Yes
    b. No

32. Where did you last got tested for an STI?
    a. At the general practitioner (GP)
    b. At the STI clinic (GGD)
    c. In the hospital
    d. At an HIV treatment center
    e. At home: with a self-sampling test that I sent to the lab for results
    f. At home: with a self-test that gave me results immediately
    g. Somewhere else, please specify…

33. Have you had an STI in the past year?
    a. Yes
    b. No

34. Which STI have you had in the past year?
    a. Chlamydia
    b. Hepatitis B
    c. LGV
    d. Hepatitis C
    e. Gonorrhea

f. HIV
g. Syphilis/lues
h. Herpes
i. Genital warts
j. Other, please specify…

35. Earlier, you have indicated that you have tested for HIV before.
Approximately when: …

36. How many times have you tested for HIV in the last two years?
Number of times: …

37. Which statement applies to you regarding testing for HIV? Single response question
    a. I test every 3 months
    b. I test every 6 months
    c. I test once a year
    d. I test every 2 or 3 years
    e. I have only tested once

38. Where did you last test for HIV?
    a. At the general practitioner (GP)
    b. At the STI clinic (GGD)
    c. In the hospital
    d. At the HIV treatment center
    e. At home: with a self-sampling test that I sent to the lab for results
    f. At home: with a self-test that gave me the result immediately
    g. Somewhere else, please specify …
    h. I would rather not say

39. You are currently applying for a self-sampling testkit of the Public Health Service (GGD). Why did you choose for a self-sampling testkit for HIV and STI?
    a. I can perform the test at my convenience
    b. I can choose where to perform the test
    c. I can perform the test without leaving home
    d. I can perform the test without running into any acquaintances
    e. I can perform the test without running into other test-site visitors
    f. I do not have to travel to the test site (saves time)
    g. I do not have to travel to the test site (saves money)
    h. I do not need to be examined by a health care provider (on test location)
    i. I do not have to talk to a health care provider (on test location)
    j. Other, please specify…

40. The STI clinic in Limburg (GGD) would like to bring self-sampling test kits to the attention of men who (also) have sex with men. Would you share this link with others?
    a. Yes
    b. Maybe
    c. No

41. With whom would you share this link?
    a. With my regular partner
    b. With my sex partner(s)
    c. With good friend(s) who are close to me

d. With other friends or acquaintances
e. With chat contacts
f. Online sharing on social media
g. With others, please specify…
h. I do not know yet with whom I want to share this link

42. Are you experiencing problems with sex or sexuality?
    a. Yes
    b. No

43. Which problems do you experience with sex or sexuality?
    a. Pain during sex
    b. Less excitement/desire for sex
    c. Erection problems
    d. Orgasmic problems
    e. Problems with sexual orientation or gender
    f. Questions about my body
    g. Other, please specify…

44. Do you have negative experiences with sex?
    a. No
    b. Yes, in the past 6 months
    c. Yes, more than 6 months ago

45. Are you fully vaccinated against Hepatitis B (with proof of titter)?
    a. I have not been vaccinated against Hepatitis B
    b. I am not sure whether I have been vaccinated against Hepatitis B
    c. I am sure I am fully vaccinated against Hepatitis B
    d. I am still in the process of getting vaccinated against Hepatitis B
    e. I have had the Hepatitis B virus in the past
    f. I have chronic Hepatitis B

46. Are you a swinger?
    a. Yes
    b. No

47. Have you had group sex in the past 6 months?
    a. Yes
    b. No

48. Have you had sex with an HIV+ partner in the past 6 months?
    a. Yes
    b. No
    c. I do not know

49. Do you know people close to you who are living with HIV?
    a. Yes
    b. No

50. Have you had sexual contacts in the last 6 months via:
    a. Swinger/couples club
    b. Sauna

c. Party at home
d. Organized party
e. Cruising
f. App (like Grindr or Tinder)
g. Website
h. Cinema
i. Holiday spot
j. Other, please specify…

51. I have sex with:
    a. Men
    b. Women
    c. Both men and women

52. How many men have you had sex with in the past 6 months?
53. How many women have you had sex with in the past 6 months?

54. What is your country of birth?
55. What is your father's country of birth?
56. What is your mother's country of birth?

57. Have you had a sexual partner from one of the following countries/areas in the last six months? Suriname / Dutch Antilles or Aruba / Morocco / Africa / Turkey / South- or Central America / Eastern Europe
    a. Yes
    b. No

58. I consider myself:
    a. Heterosexual
    b. Homosexual/Gay
    c. Bisexual
    d. None of the above

59. Who did you have sex with in the past six months?
    a. Casual partner(s)
    b. Steady partner(s)
    c. Both regular and steady partner(s)

60. Are/have you been in a relationship with this steady sex partner?
    a. Yes
    b. No

61. Did you have sex with someone else during your relationship?
    a. Yes
    b. No

62. Did your sex partner have other sex contacts during the relationship?
    a. Yes
    b. No
    c. I do not know

63. When was your last sexual contact with a steady sex partner?

a. In the past 2 weeks
b. In the past month
c. In the past 2 months
d. In the past 3 months
e. More than 3 months ago

64. This steady partner is a:
    a. Man
    b. Woman
    c. Transgender
    d. None of the above

65. Is this your ex-partner?
    a. Yes
    b. No

66. In the past 6 months, did you have vaginal sex with a steady sex partner?
    a. No
    b. Yes, always with a condom
    c. Yes, sometimes with a condom
    d. Yes, never with a condom

67. In the past 6 months, did you have oral sex with a steady sex partner?
    a. No
    b. Yes, always with a condom
    c. Yes, sometimes with a condom
    d. Yes, never with a condom

68. In the past 6 months, did you receive anal sex from a steady sex partner?
    a. No
    b. Yes, always with a condom
    c. Yes, sometimes with a condom
    d. Yes, never with a condom

69. In the past 6 months, did you give anal sex to a steady sex partner?
    a. No
    b. Yes, always with a condom
    c. Yes, sometimes with a condom
    d. Yes, never with a condom

70. When was your last sexual contact with a casual sex partner?
    a. In the last 2 weeks
    b. In the last month
    c. In the last 2 months
    d. In the last 3 months
    e. More than 3 months ago

71. This casual partner is a:
    a. Man
    b. Women
    c. Transgender
    d. None of the above

72. In the past 6 months, did you have vaginal sex with a casual sex partner?
    a. No
    b. Yes, always with a condom
    c. Yes, sometimes with a condom
    d. Yes, never with a condom

73. In the past 6 months, did you have oral sex with a casual sex partner?   No
    a. Yes, always with a condom
    b. Yes, sometimes with a condom
    c. Yes, never with a condom

74. In the past 6 months, did you receive anal sex from a casual sex partner?
    a. No
    b. Yes, always with a condom
    c. Yes, sometimes with a condom
    d. Yes, never with a condom

75. In the past 6 months, did you give anal sex to a casual sex partner?
    a. No
    b. Yes, always with a condom
    c. Yes, sometimes with a condom
    d. Yes, never with a condom

76. Have you received money or gifts in exchange for sex?
    a. Yes, in the past 6 months
    b. Yes, more than 6 months ago
    c. No

77. Where have you done sex work in the past 6 months?
    a. At home, regular clients
    b. At home, clients via advertisements online
    c. Car date
    d. Escort (agency)
    e. (Sauna) club
    f. Brothel
    g. Party
    h. Other, please specify…

78. Did you pay with money or goods in exchange for sex?
    a. Yes, in the last 6 months
    b. Yes, more than 6 months ago
    c. No

79. Do you use anal douches (enemas)?
    a. Yes
    b. No
80. Regardless of your STI test results, do you plan to get tested again this year?
    a. Definitely not
    b. Probably not
    c. Neutral

   d. Probably
   e. Definitely

81. If I want to get tested for an STI and HIV in the next 6 months, I would:
    a. Go to the general practitioner (GP)
    b. Go to the STI clinic (GGD)
    c. Go to the hospital
    d. Go to the HIV treatment center
    e. Test at home: with a self-sampling test that I send to the lab for results

82. The following questions are about remote consultations. In a remote consultation you talk to or get support from a nurse or doctor (of the STI clinic of the GGD). These remote consultations can take place online or via phone.
    If you could have a remote consultation, would you want to?
    a. Yes, I am open to this
    b. No, I prefer a consultation on a test location
    c. No, I do not want a consultation (at this moment)

83. If you could have a remote consultation, which method of communication would you prefer the most?
    a. Telephone consultation
    b. Online consultation (via chat)
    c. Webcam consultation (via video call)

84. During a remote consultation, what topics would you like to discuss? You can indicate multiple answers.
    a. Safer sex to prevent HIV and STIs
    b. Use of PrEP
    c. Ways of HIV and STI testing
    d. Dealing with a positive HIV test result (if you have HIV)
    e. Dealing with positive STI test results (if you have an STI)
    f. My sexuality (e.g., problems with erection, arousal or ejaculation)
    g. My body (e.g., genitals)
    h. My orientation
    i. Enjoyment of sexual contact
    j. Drug use during sex
    k. Vaccination for Hepatitis B
    l. Unwanted sexual experiences
    m. Dealing with social sexual pressure
    n. Use of dating apps, websites or social media
    o. Other, please specify

85. Would you like to have a conversation with a health care professional? The health care professional will contact you by telephone call.
    a. Yes, I would like to
    b. No, I do not have further questions

86. Do you have any questions or comments? Please let us know below.

87. Would you like to receive the package at home of rather pick up at a central location?
    Receive at home
    Pick-up

88. We ask you to enter your personal information below:

In order to provide you with a good service, we need this information. They will be treated strictly confidentially. All our employees have professional confidentiality. No information about tests and results will go to your doctor, insurance or others, unless you have given explicit permission. The data can be used to contact you in case of a positive result.

a. First name
b. Last name
c. Date of birth
d. Postal code
e. Street
f. Number
g. Addition
h. City
i. E-mail address
j. Cell phone number

89. How can we contact you in case of negative test results (no STI)?
a. Via a text message (SMS)
b. By phone (calling)
c. Via an e-mail

Click "next" to send us your application. Your application will then be processed, and we will send you the self-sampling testkit.

Do you have any questions? For more information, please visit our website www.limburg4zero.nl or e-mail us via limburg4zero@ggdzl.nl
