# Supplementary material for: Evaluating the Implementation of Home-Based Sexual Health Care Among Men Who Have Sex with Men: Limburg4zero
Source: AIDS Behav. 2025 Jan 8;29(3):976–92. doi: 10.1007/s10461-024-04579-6 (PMC11830641; doi:10.1007/s10461-024-04579-6)
Supplement: Supplementary file 3 — Supplementary file3 (PDF 372 kb)—Evaluation questionnaire Limburg4zero [file 10461_2024_4579_MOESM3_ESM.pdf]

Evaluating the implementation of home-based sexual health care among men who have sex with men; Limburg4zero. Goense*, Evers, Manait, Hoebe, van Loo, Posthouwer, Ackens, van Hooren, Theuerzeit, Crutzen, Stutterheim, and Dukers-Muijrers; *c.goense@maastrichtuniversity.nl

Supplementary material S3. Evaluation questionnaire Limburg4zero

1. If you participate in the study, we ask you to give informed consent, to store, and use your data for this study. When analyzing and reporting the research study, your data cannot be traced back to you. If you give informed consent to participate and/or use of data, unfortunately you cannot participate in the study.
   a. Yes, I give informed consent to participate in the underlying study and to be linked to data from previous research.
   b. Yes, I give informed consent to the storage and use of my answers for underlying research. When analyzing and reporting the research, my data cannot be traced back to me.

2. What was a reason for you to do a Sexually Transmitted Infection (STI) test?
   a. I want to prevent infection of partner(s)
   b. For my own health
   c. Because I suffer from symptoms
   d. For periodic monitoring
   e. I have been warned
   f. I‘ve been invited in the pilot and want to help improve sexual health care
   g. Other, namely…

3. The following statements are about the questionnaire you completed to apply for the self-sampling testkit. Please indicate whether you agree with the statements below:
   1) Strongly disagree, 2) Disagree, 3) Neither agree nor disagree,4) Agree, 5) Strongly agree

   a. I understood all questions asked in the questionnaire
   b. There were questions I didn‘t want to answer
   c. Completing the questionnaire took me a long time

4. How can we make these questions easier to understand?

5. The following questions are about the self-sampling testkit
   To what extent are you satisfied with ...
   1) Totally disagree, 2) Disagree, 3) Neither agree nor disagree,4) Agree, 5) Totally agree

   a. The time between signing up and receiving the package

   b. Anonymity of the package
   c. Instructions in the package
   d. The information letter in the package
   e. The information on the website (www.limburg4zero.nl)
   f. The instruction video
   g. Time it takes to run the tests
   h. Returning the self-sampling test (by mail)
   i. Time between returning and getting the results
   j. The opportunity to talk with a HCP of the STI clinic

6. You indicated to disagree with: [XXX] How can we improve this?

7. How clear did you find the information letter?
   a. Unclear
   b. Reasonably understandable
   c. Clear

8. How clear did you find the instructions for the throat, urine, anal, and blood test?
   1) Unclear, 2) Reasonably understandable, 3) Clear
   a. Throat
   b. Urine
   c. Anal
   d. Blood

9. Can you indicate what you found unclear about …
   a. Information letter
   b. Instructions for the throat test
   c. Instructions for the urine test
   d. Instructions for the anal test
   e. Instructions for the blood test

10. How easy did you find performing the throat, urine, anal, and blood test? (scale)
   (0. Easy - 100. Difficult)
   a. Throat
   b. Urine
   c. Anal
   d. Blood

11. Could you indicate per test what you found difficult?
   a. Throat
   b. Urine
   c. Anal
   d. Blood

12. What do you think can be improved about the self-sampling test kit? …

13. Have you been in contact with the STI clinic? For example, before or after performing a self-sampling test kit?
   a. Yes
   b. No
   c. Not yet, but I intend to

14. In what ways have you been in contact with the STI clinic?
   a. By phone
   b. On site
   c. By e-mail (limburg4zero@ggdzl.nl)
   d. By text message (SMS)

15. Please indicate whether you agree with the following statements:
   1) Totally disagree, 2) Disagree, 3) Neither agree nor disagree,4) Agree, 5) Totally agree

a. The employee took enough time for me
b. I understood what the employee said/wrote
c. I was given plenty of opportunity to ask questions
d. I felt safe
e. The employee explained everything clearly

16. To what extent are you satisfied with your contact with the STI clinic?
    a. Totally disagree
    b. Disagree
    c. Neither agree nor disagree
    d. Agree
    e. Totally agree

17. Have you visited the website www.limburg4zero.nl?
    a. Yes
    b. No
    c. Not yet, but I intend to

18. Did you find the information you were looking for?
    a. Yes
    b. No, in fact I was looking for information about ...

19. What do you think could be improved about the website? The website is still under development, so we would love to hear opinions and tips about the website www.limburg4zero.nl

20. Did you watch the instructional video before or during testing?
    a. Yes
    b. No

21. What did you think of the instructional video?
    1) Totally disagree, 2) Disagree, 3) Neither agree nor disagree,4) Agree, 5) Totally agree

    a. The instructional video helped me complete the tests
    b. The instructional video was difficult to understand
    c. The instructional video gives a realistic picture of how to complete the tests
    d. The instruction video did not have additional value for me
    e. I can identify with the person who is performing the tests
    f. The instructional video was clear

22. In addition to the self-sampling test kit, did you need additional support?
    a. Yes, for practical issues
    b. Yes, for mental support
    c. No

23. On what topics did you need support?
    a. Having safer sex to prevent HIV and STI
    b. Use of PrEP
    c. Methods of HIV and STI testing
    d. Dealing with a positive HIV test result (if you have HIV)
    e. Dealing with a positive STI test result (if you have a STI)
    f. My sexuality (e.g.: problems with erection, arousal, or ejaculation) sexual arousal

g. My body (e.g.: genitals)
h. My orientation
i. Pleasure in sexual contact
j. Use of drugs during sex
k. Vaccination for Hepatitis B
l. Unwanted sexual experiences
m. Dealing with social pressure regarding sexuality
n. Using dating apps, websites or social media
o. Other, namely…

24. How would you like to receive this support?
    a. By written information on a website
    b. By a video or film on the website
    c. By an online chat service with a healthcare professional
    d. By a webcam interview with a healthcare professional
    e. By a telephone conversation with a healthcare professional
    f. Face-to-face conversation with a healthcare professional
    g. Through a friend/sex partner/acquaintance who can explain this to me
    h. Otherwise, namely…

25. How likely is it that you would recommend the self-sampling testkit to others? (scale)

0. Very unlikely - 100. Very likely

26. Who would you recommend the self-sampling testkit?
    a. To my steady relationship
    b. To my sex partner(s)
    c. To good friend(s) who are close to me
    d. To other friends or acquaintances
    e. To chat contacts
    f. I would share a post on social media
    g. To others, namely…
    h. I don‘t know yet who I would recommend self-sampling testkit
    i. I would not recommend it to anyone

27. What are reasons for you to recommend the home-sampling test kit not to others?
    a. I feel ashamed / I don‘t dare
    b. Testing for STI/HIV is private for everyone
    c. I think they already test regularly for STI/HIV
    d. I think they are at no/little risk for STI/HIV
    e. I don‘t think they are able to take these tests themselves
    f. I don‘t know anyone (to whom I can give the test)
    g. Other, namely…
    h. None, I would recommend the home test however

28. What are reasons for you to recommend the self-sampling testkit well to others?
    a. To protect their health
    b. If they have been warned for an STI
    c. This is handy if they experience complaints
    d. If they want to test periodically
    e. Otherwise, namely…
    f. None, I would not recommend the self-sampling testkit

29. How often do you discuss HIV and STI related topics with others? (scale)
    1.Never - 5. Very often

30. When you discuss HIV and STI related topics with others, how much information do you provide? (scale)
    1.Almost none - 5. A lot of

31. In the past 3 months, with how many people have you discussed HIV and STI related topics? (scale)
    1.None - 5. A lot

32. How likely are others in your social network to ask you for advice about HIV/STIs? (scale)
    1.Very unlikely - 5. Very likely

33. When discussing HIV/STIs with others in your network, which scenario is most likely? (scale)
    1.You always give others information - 5. Others always give you information

34. When you discuss HIV/STIs with others, how often do they ask you for advice? (scale)
    1.Never - 5.Very often

35. If you would like to test for HIV/STI again in the future, would you use the self-sampling test kit again?
    a. Definitely not
    b. Probably not
    c. Neutral
    d. Probably yes
    e. Definitely yes
36. Can you explain this?...

37. If you could choose, in what way would you prefer to be tested at the STI clinic?
    a. I would like all testing by means of a self-sampling testkit
    b. I would prefer some testing by means of a self-sampling testkit and some on location: a combination of remote and on-site testing
    c. I wouldn‘t prefer a self-sampling testkit (anymore): all testing on site

38. If you could choose, in what way would you prefer to be offered consultations at the STI clinic?

    a. I would like all consultations online/by telephone
    b. I would prefer some consultations online and some on location: a combination of remote and on-site testing
    c. I would not prefer online/by telephone consultations: all on-site consultations

39. Finally, please rate the self-sampling testkit of Limburg4Zero: *Rating 1 to 10.*

40. The STI clinic regularly conducts research to improve their care. Whether it's filling out a questionnaire, participating in a focus group, or a focus group, your participation is very valuable. May we invite you again in the future?
    a. Yes, *E-mail address*
    b. No

41. The STI clinic of the Center for Sexual Health wants to stay informed about developments regarding sexuality and STI/HIV testing so that care is connected. Can you tell us anything about this? If you have any comments after completing the questionnaire, you can always email limburg4zero@ggdzl.nl
