# Supplementary material for: Evaluating the Implementation of Home-Based Sexual Health Care Among Men Who Have Sex with Men: Limburg4zero
Source: AIDS Behav. 2025 Jan 8;29(3):976–92. doi: 10.1007/s10461-024-04579-6 (PMC11830641; doi:10.1007/s10461-024-04579-6)

Evaluating the implementation of home-based sexual health care among men who have sex with men; Limburg4zero. Goense*, Evers, Manait, Hoebe, van Loo, Posthouwer, Ackens, van Hooren, Theuerzeit, Crutzen, Stutterheim, and Dukers-Muijrers; *c.goense@maastrichtuniversity.nl

Supplementary material S4. Communication material for promoting Limburg4zero among MSM in the Limburg region of the Netherlands

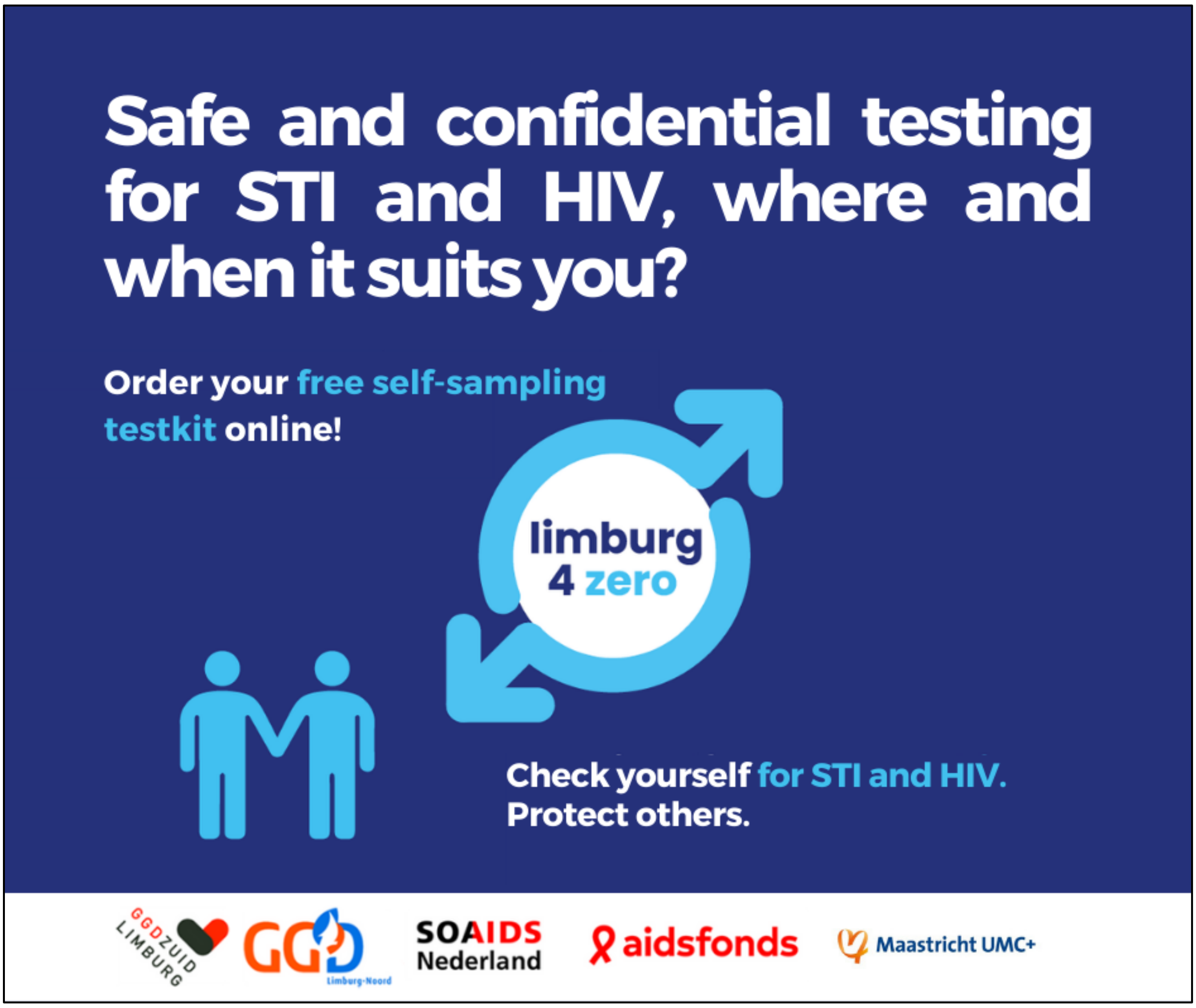

Supplement: Supplementary file 4 — Supplementary file4 (PDF 198 kb)—Communication material for promoting Limburg4zero among MSM in the Limburg region of the Netherlands [file 10461_2024_4579_MOESM4_ESM.pdf]
