# Supplementary material for: Evaluating the Implementation of Home-Based Sexual Health Care Among Men Who Have Sex with Men: Limburg4zero
Source: AIDS Behav. 2025 Jan 8;29(3):976–92. doi: 10.1007/s10461-024-04579-6 (PMC11830641; doi:10.1007/s10461-024-04579-6)
Supplement: Supplementary file 5 — Supplementary file5 (PDF 154 kb)—Characteristics participants semi-structured interviews with HCP (n=14) [file 10461_2024_4579_MOESM5_ESM.pdf]

Evaluating the implementation of home-based sexual health care among men who have sex with men; Limburg4zero. Goense*, Evers, Manait, Hoebe, van Loo, Posthouwer, Ackens, van Hooren, Theuerzeit, Crutzen, Stutterheim, and Dukers-Muijrers; *c.goense@maastrichtuniversity.nl

Supplementary material S5. Characteristics participants semi-structured interviews with HCPs (n=14)

Characteristics	HCPs (n=14)
**Gender**	
Male	4
Female	10
**Age**	
20-30	3
31-40	5
41-50	2
50 >	4
**Work environment**	
Centre for Sexual Health	7
GP office	7
**Work type**	
Sexual health doctor	2
Sexual health nurse	5
GP	7
**Work experience (current)**	
0-10 years	6
11-20	4
20>	1
Unknown	3
**Frequency consultations (MSM)**	
Daily	6
Weekly	2
Monthly	1
Unknown	5
